# Precessional Dynamics of Octahedra in CsPbBr_3_

**DOI:** 10.1021/acs.jctc.5c02166

**Published:** 2026-04-16

**Authors:** Lucas Martin Farigliano, Marcio S. Gomes-Filho, Alexandre Reily Rocha, Gustavo Martini Dalpian

**Affiliations:** † Departamento de Física dos Materiais e Mecânica, Instituto de Física, 74362 Universidade de São Paulo, São Paulo, São Paulo 05508-090, Brazil; ‡ Departamento de Química Teórica y Computacional, Facultad de Ciencias Químicas, INFIQC, CONICET, Universidad Nacional de Córdoba, Córdoba X5000HUA, Argentina; § Centro de Ciências Naturais e Humanas, 425753 Universidade Federal do ABC, Santo André, São Paulo 09210-580, Brazil; ∥ Institute of Theoretical Physics, 133637 São Paulo State University, Campus São Paulo, São Paulo 01140-070, Brazil

## Abstract

The crystal structure of ABX_3_ halide perovskites consists of corner-sharing BX_6_ octahedra, whose collective distortions define the different crystallographic phases. Because these materials are mechanically soft, with a shallow energy landscape, the octahedra display pronounced dynamical fluctuations that play a key role in determining their structural transitions and physical properties. Directly resolving this motion is challenging: experimentally, the dynamics occur on time scales that elude conventional spectroscopic techniques, while ab initio molecular dynamic is computationally prohibitive for the system sizes required. Here, we develop a machine-learning potential that enables large-scale molecular dynamics simulations with near-DFT accuracy. We follow the temporal evolution of individual octahedra in CsPbBr_3_ and identify the characteristic events that define their rotational behavior. Our simulations reveal that the commonly assumed tilting sequence—where an octahedron switches from a positive tilt to an untilted configuration, and then to a negative tilt (the *a* ^+^ ⇒ *a* ^0^ ⇒ *a* ^–^ pathway in Glazer notation)—rarely occurs under the specific conditions investigated in this study. Instead, the dominant mode of motion arises from a combined precession–nutation process in which the octahedron continuously reorients around its axis while undergoing smaller-amplitude oscillations. These findings suggest that similar complex rotational dynamics should be expected across halide perovskites, offering new microscopic insight into their structural behavior and guiding future experimental efforts aimed at detecting these motions.

## Graphical abstract

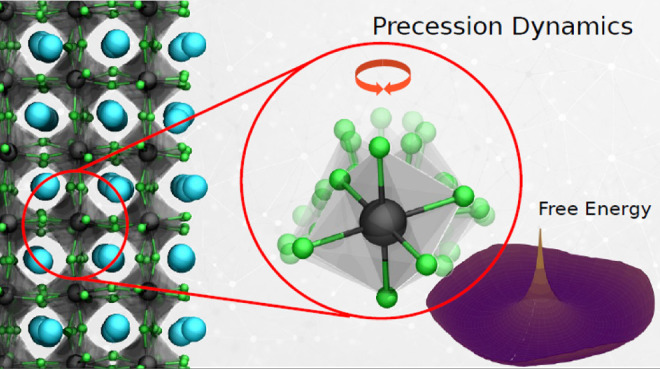

## Introduction

Halide perovskites exhibit outstanding physical and chemical properties, including high defect tolerance and superior optical characteristics compared to other materials. Moreover, they can be synthesized through relatively simple, efficient, and low-cost methods.^1,2^ In recent years, perovskite-based solar cells have achieved a certified efficiency of 26.95% ^3,4^ positioning themselves as one of the most promising photovoltaic technologies. Simultaneously, the performance of perovskite-based light-emitting diodes (LEDs) has steadily improved since 2014, reaching external quantum efficiencies above 20% for green, red, and near-infrared emission, and attaining values close to 18% for blue emission.^5–8^ These advancements have not only driven progress in photovoltaics and lighting but have also expanded the potential of halide perovskites into emerging applications such as X-ray detectors, field-effect transistors, and lasers.^9–12^

They are typically described by the general formula ABX_3_, consisting of a three-dimensional network of corner-sharing [BX_6_]^4–^ octahedra. The connectivity and distortion (tilting and rotation) of these octahedra play a decisive role in determining the material’s electronic structure, optical absorption, and charge transport properties. These materials are further distinguished by a soft crystal lattice, which imparts high defect tolerance and enables them to accommodate substantial structural disorder.^13^ Such behavior arises from the pronounced anharmonicity of their lattice vibrations, which enhances structural flexibility. This anharmonicity has been shown to facilitate polaron formation, improve charge carrier dynamics, and contribute to both a strong electrostrictive response and an ultralow thermal conductivity.^14^ Furthermore, even subtle structural modifications within the metal–halide framework, particularly variations in octahedral tilting, can induce substantial changes in the functional properties of the material.^15–17^

These distortions can be induced by a range of intrinsic and extrinsic factors. For example, the inclusion of oversize A-site cations may disrupt the 3D lattice and promote the formation of lower-dimensional perovskite phases, typically associated with wider band gaps and enhanced exciton binding energies. Likewise, external stimuli such as temperature and pressure are known to trigger phase transitions that reorganize octahedral connectivity, thereby affecting carrier mobility and recombination dynamics.^18,19^ Furthermore, chemical attributes like electronegativity can modulate the bonding environment within the lattice, offering an additional handle to tune the octahedral configuration and, consequently, the material’s optoelectronic response.^20,21^

Traditionally, octahedral distortions are analyzed using the Glazer notation or its derivatives, which systematically describe the tilting and rotation modes of octahedra.^22–24^ This type of nomenclature is particularly useful in static calculations, such as those based on density functional theory (DFT), to identify average configurations that adequately represent the experimentally observed crystal structure, for instance, through X-ray diffraction. A representative example of the use of Glazer’s notation is provided by the work of Li et al.,^23^ who analyze octahedral behavior by examining tilt patterns along Cartesian directions. By combining machine learning techniques with DFT calculations, the authors approximate the potential energy surface associated with various distortions, offering key insights into the structural stability of perovskites. Similarly, Bechtel and Van der Ven^24^ employ *ab initio* calculations to explore the Born–Oppenheimer energy surface in terms of symmetrized order parameters, enabling the identification of structural instabilities associated with octahedral distortions in perovskites of the form CsMX_3_ (M = Pb or Sn; X = Br or I).

These approaches, however, generally assume symmetric distortion patterns, effectively treating octahedral tilts and rotations as collective modes that preserve both the ideal octahedral geometry and the overall symmetry of the material. As a consequence, they do not explicitly account for local disorder in the crystal lattice, thereby excluding the instantaneous configurations accessible in molecular dynamics simulations. Although this framework has been highly successful in rationalizing structural trends, it is not sufficient to fully capture the complexity of these systems. For instance, it is well established that at elevated temperatures most ABX_3_ perovskites—whether hybrid or fully inorganic—adopt the ideal cubic structure type with space group *Pm*3̅*m*, a high-symmetry phase characterized by a single lattice parameter. It is also well accepted nowadays that this cubic structure emerges as a time average of distorted configurations, often called polymorphous structures.^25^ This representation naturally introduces local disorder and leads to electronic properties in closer agreement with experimental measurements, indicating that octahedral distortions do not strictly follow symmetric patterns and that these materials exhibit a highly anharmonic character—a regime in which Glazer’s notation loses much of its descriptive power.

To better capture these distortions, several studies have implemented molecular dynamics (MD) simulations, using both *ab initio* descriptions^26–29^ and classical potentials, including those generated via machine learning techniques.^30–38^ These approaches enable a detailed exploration of the atomic dynamics within the system. Nevertheless, significant uncertainties remain regarding the time evolution of octahedral distortions. One of the key open questions concerns the actual trajectories followed by octahedra as they fluctuate over time. Only a few studies have examined tilting behavior at different temperatures using MD simulations,^30,34^ typically by tracking the inclination of the vector connecting the halogen atoms to the central atom of the octahedron to characterize its evolution across the different phases that dominate each temperature regime.

In this work, we evaluate the dynamics of octahedra using an angular description, based on molecular dynamics simulations driven by our high-accuracy machine-learning potentials, with CsPbBr_3_ selected as a model system due to its rich structural dynamics and its relevance in optoelectronic applications. To quantify octahedral behavior, we employ a set of spherical-coordinate-based descriptors that capture the orientation of each octahedron in three-dimensional space. These angular variables enable a detailed analysis of structural distortions and their temporal evolution, allowing us to identify distinct motional mechanisms. In particular, we study precessional dynamics—characterized by the evolution of the azimuthal angle φ—which describe rotations of the octahedron around its principal axis, and nutational dynamics—captured through variations in the polar angle θ—which correspond to oscillations in the tilt amplitude.

Our analysis reveals that precessional and nutational motions, shown in Figure 1a, play a dominant role in octahedral dynamics, demonstrating that their combined action provides a more faithful description of octahedral motion than the commonly assumed, but oversimplified, picture in which the tilting dynamics follow a sequence *a* ^+^ ⇒ *a* ^0^ ⇒ *a* ^–^ in Glazer notation (Figure 1b). Such a sequential mechanism is shown to be highly unlikely to occur.

**Figure 1:**
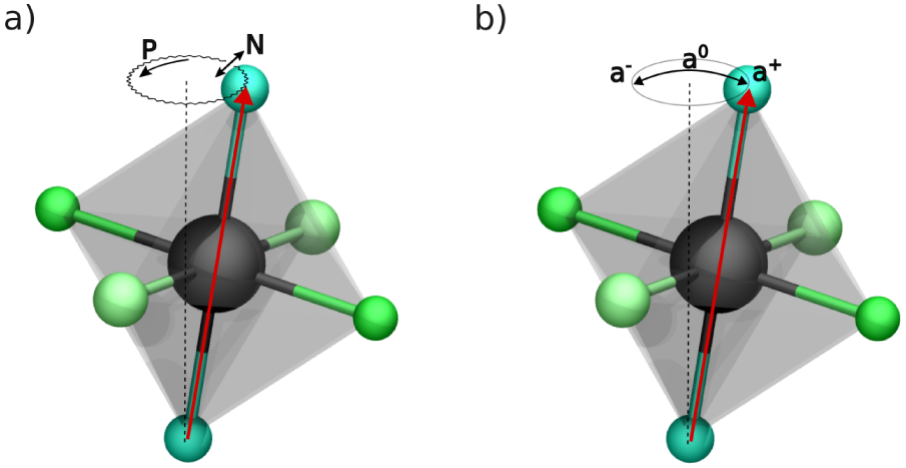
Comparison of octahedral dynamic mechanisms in CsPbBr_3_. a) Representation of the primary angular dynamics, showing precession (P, changes in the azimuthal angle φ) and nutation (N, oscillations in the polar angle θ) as combined motion mechanisms. b) Schematic representation of the simplified, commonly assumed sequential tilting mechanism, where the tilt follows the sequence *a* ^+^ ⇒ *a* ^0^ ⇒ *a* ^–^ in Glazer notation.

Although our analysis is made specifically for CsPbBr_3_, considering that many other halide perovskites share the same soft dynamic lattice behavior, we believe that our findings should also be relevant for other compounds in this family.

## Theoretical Methods

We developed a highly accurate deep neural network interatomic potential for the CsPbBr_3_ perovskite using the GPU-accelerated DeePMD-kit package.^39–41^ The potential was constructed employing the Deep Potential–Smooth Edition (DP-SE) descriptor,^42^ which expands the local atomic environment within a smooth cutoff radius of 9.0 Å (with a smoothing region starting at 1.5 Å). The descriptor architecture used embedding networks with neuron layers of sizes [25, 50, 100], and 16 axis neurons, with selection numbers of [32, 32, 64] for Cs, Pb, and Br, respectively.

The fitting network consisted of three fully connected layers with 240 neurons each, employing residual connections to improve training stability. Model optimization followed an exponential learning rate schedule, starting from 1 × 10^–3^ and decaying to 3.51 × 10^–8^ over 10,000 decay steps. The loss function combined contributions from energies and forces, with an initial weighting of 0.02 for the energy term and 1000 for the force term, gradually adjusted to final values of 1 for both. This parametrization strategy ensures that the resulting potential accurately reproduces reference energies and forces, while maintaining transferability to a wide range of structural environments relevant to CsPbBr_3_ under different thermodynamic conditions, similar to other works.^43–45^

The training process follows a concurrent learning scheme.^46,47^ It consists of four main stages: *(i) Initial committee training:* A preliminary data set is used to train a committee of four independent DP models. Each model in the committee shares the same network architecture and training data but is initialized with a different random seed. This ensemble approach enables the estimation of model uncertainty and ensures robustness during subsequent active learning stages. *(ii) Exploration:* The trained DP models are combined in a committee to perform deep potential molecular dynamics (DPMD) simulations under varying thermodynamic conditions (different pressures and temperatures). Configurations where the four models exhibit the maximum standard deviation in atomic forces are identified as unexplored regions of the potential energy surface. *(iii) Labeling:* The selected configurations are evaluated using *ab initio* calculations to obtain energy and force values. These newly labeled configurations are then added to the data set for subsequent refinement. *(iv) Final Training:* A new set of four DP models is trained using the updated data set, which incorporates newly labeled configurations. This iterative process (steps ii-iv) is repeated until the criterion of convergence is achieved, providing a robust DP model with fewer data points and improved accuracy.

To construct the DP model for CsPbBr_3_, we initially used an *ab initio* NPT molecular dynamics data set from previous work.^27^ In this study, phase transitions in CsPbBr_3_ perovskite were investigated over a temperature range from 150 to 500 K, with pressure held constant at zero. The calculations were performed at the DFT level^48^ using the CP2K software,^49^ employing the PBEsol exchange-correlation functional^50^ and DZVP molecularly optimized basis functions.^51^ The system was modeled using a 4 × 4 × 4 supercell containing 320 atoms. To explore the phase transitions we employed two distinct methodologies: the temperature ramp method, in which the system was initially equilibrated at 600 K and then gradually cooled, with each new simulation starting from a thermally equilibrated configuration from the previous step; and the constant temperature method, where independent simulations were performed at fixed temperatures (80–100 ps per simulation), starting from a frozen 0 K cubic structure.

The initial DP training data set was constructed from 1162 configurations randomly selected from the temperature-ramp trajectories. In addition, 472 of these configurations were further compressed by approximately 5% to improve the sampling of short-range interatomic distances. We also incorporated 0 K configurations of the cubic, orthorhombic, and tetragonal phases, as well as configurations obtained by compressing or expanding the fully relaxed structures by about 5% relative to the most stable structure.

To improve sampling near the orthorhombic–tetragonal–cubic transition regions, additional configurations were generated in the temperature range 300–375 K. Specifically, we augmented the training set with 408 configurations obtained from AIMD simulations at 300, 325, 350, 375 K.

The initial training data set was then used within a concurrent learning framework, as described above. The DP models were evaluated across a range of thermodynamic conditions. For each pressure (0, 1, 10, 100, 1000 bar), independent simulations were performed at target temperatures ranging from 200 to 500 K in 20 K increments. The maximum atomic force error across the committee models was monitored over 100 ps of simulation. Configurations exhibiting errors between 0.1 and 0.3 eV/Å were selected for inclusion in the training set.^47^ In total, 153 such configurations were identified, for which DFT calculations were performed, followed by retraining of the models (first iteration). In the second iteration, no additional configurations within the specified error range were identified, indicating that the model is accurate.

In this manner, the final DP models—constructed from the initial training data set, configurations suggested through active learning—are capable of accurately reproducing the stable phases of CsPbBr_3_. In the Supporting Information (SI), Figure S1 presents parity plots comparing DP-predicted energies and forces with their DFT counterparts, demonstrating the high fidelity of the model. We found that the final DP model performs well on the train data set, with the root mean squared error (RMSE) for energy equal to 0.32 meV/atom and 24 meV/Å for forces.

To assess the performance of the DP model, we carried out two types of tests. First, we evaluated the model on a separate test data set, composed of 176 configurations (approximately 20 snapshots per temperature, as indicated in Figure 2), extracted from constant-temperature simulations^27^ and not used during training. Second, we monitored the evolution of the lattice parameters as a function of temperature and compared them with the results reported in ref 27. In Figure 2a,b, we present the parity plots for energies and forces predicted by the DP model (*E*_*NN*_ and *F*_*NN*_, respectively) against the corresponding DFT values (*E*_*DFT*_ and *F*_*DFT*_, respectively). Figure 2c,d show the comparison between the lattice vectors and the interaxial angles obtained from the DP model and those reported in ref 27.

**Figure 2:**
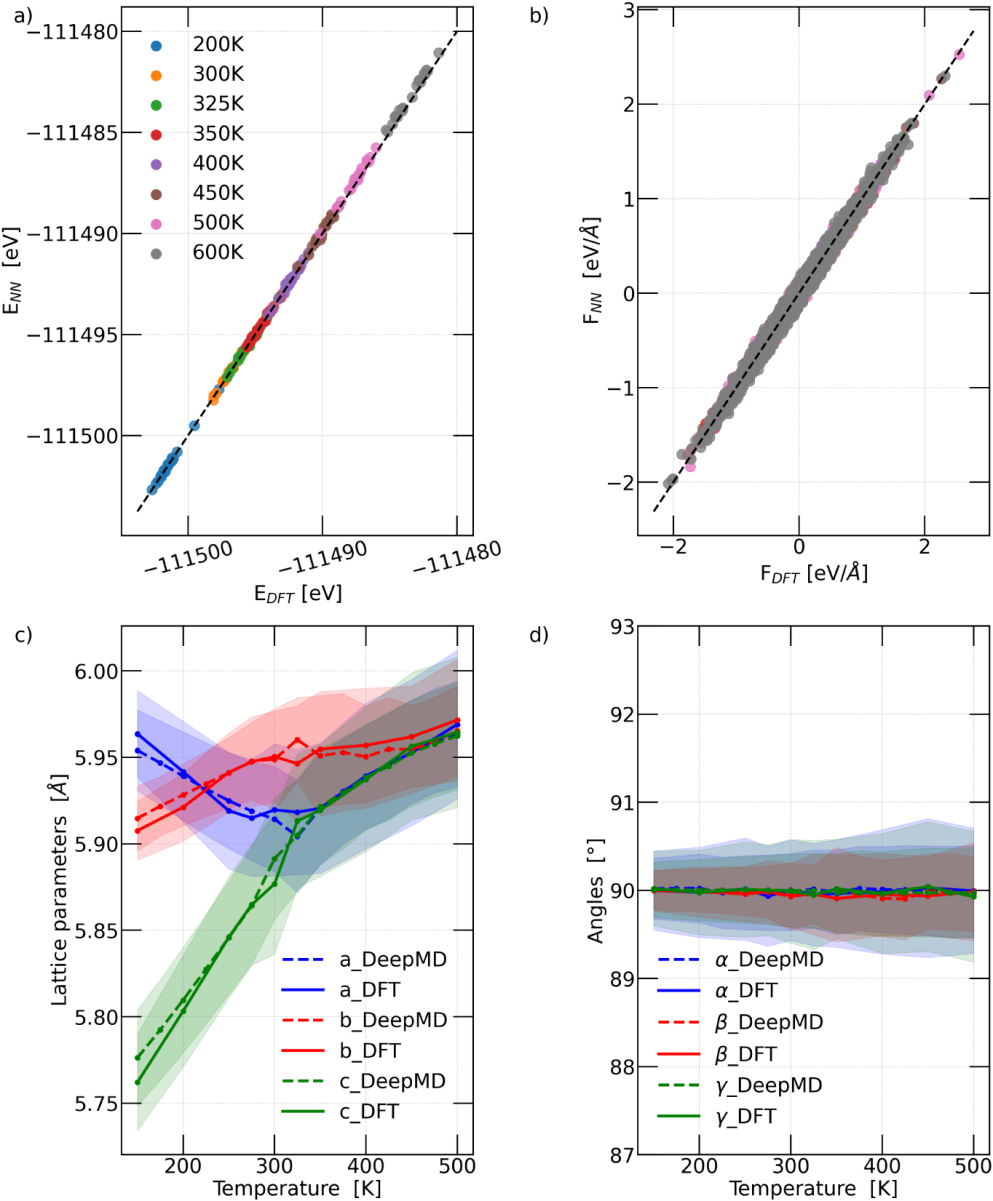
Deep neural network potential quality assessment: a) Potential energy and b) the forces predicted by the neural network (*E_NN_* and *F_NN_*) compared to the DFT reference values (*E_DFT_* and *F_DFT_*). c) and d) evaluate the potential’s accuracy in capturing the phase transitions of CsPbBr_3_, using AIMD simulation results as a reference. Specifically, panel c) illustrates the evolution of the lattice parameters (*a*, *b*, and *c*), while panel d) depicts the variation of the angles between lattice vectors (α, β, and γ) as a function of temperature for CsPbBr_3_.^27^ In c) and d), the shaded regions represent the standard deviation of the lattice parameter and angle variations with temperature, respectively.

The evaluation results indicate that the final DP model achieves high accuracy on the test data set, yielding a root mean squared error (RMSE) of 0.47 meV/atom for energies and 26 meV/Å for forces. Such low errors confirm the reliability of the potential and are consistent with the accuracy reported for high-quality deep potential models.^52^

Molecular dynamics simulations with our interatomic potential were performed using the LAMMPS package^53^ with the developed DP model. Simulations were carried out in the isothermal–isobaric (*NPT*) ensemble, employing periodic boundary conditions in all three directions. Temperature was controlled with a Langevin thermostat (damping parameter 100 fs), and pressure was maintained at 0 bar using a fully anisotropic (tri) Nosé–Hoover barostat (damping parameter 1000 fs). The equations of motion were integrated with a time step of 1 fs.

Simulations were performed over a wide temperature range, specifically at 150, 200, 225, 250, 275, 300, 325, 350, 400, 450, 500, 550, and 600 K. Two system sizes were considered: a 4 × 4 × 4 supercell (320 atoms) for direct comparison with DFT, and a 8 × 8 × 8 supercell (2560 atoms) to analyze temperature-dependent properties. Each simulation was first equilibrated for 100 ps, followed by a production stage. The production stage lasted 100 ps for the 4 × 4 × 4 supercell and 1 ns for the 8 × 8 × 8 supercell. Using the 4 × 4 × 4 supercell, we performed a direct comparison with AIMD, obtaining excellent agreement in reproducing the phase-transition temperatures of CsPbBr_3_, as illustrated in Figure 2c,d.

In order to extract meaningful structural information from the atomic configurations, we employed a geometric representation of the system based on the orientation of individual octahedra. To this end, we describe their orientation using spherical coordinates, as illustrated in Figure 3. We defined three orientation vectors, **r**_*x*_, **r**_*y*_, and **r**_*z*_, each connecting a pair of opposite Br atoms through the central Pb atom. From these vectors (shown in red), we define angular coordinates θ and φ for each Cartesian direction, grouped as θ_*x*_, θ_*y*_, θ_*z*_, φ_*x*_, φ_*y*_, φ_*z*_. The spherical angular variables were obtained from atomic coordinates expressed in the instantaneous simulation cell, whereas the angular reference frame was fixed to the Cartesian axes (*x*, *y*, *z*) and remained constant throughout the trajectory. This definition provides a consistent separation between lattice deformations and intrinsic octahedral rotational dynamics. These structural descriptors were computed from molecular dynamics trajectories of the larger 8 × 8 × 8 supercell (2560 atoms), providing a comprehensive sampling of the system’s structural fluctuations and capturing the octahedral dynamics across different phases and temperatures. To clarify the definition of these descriptors, Figure 3 illustrates a single octahedron in CsPbBr_3_ along with its associated spherical coordinates.

**Figure 3:**
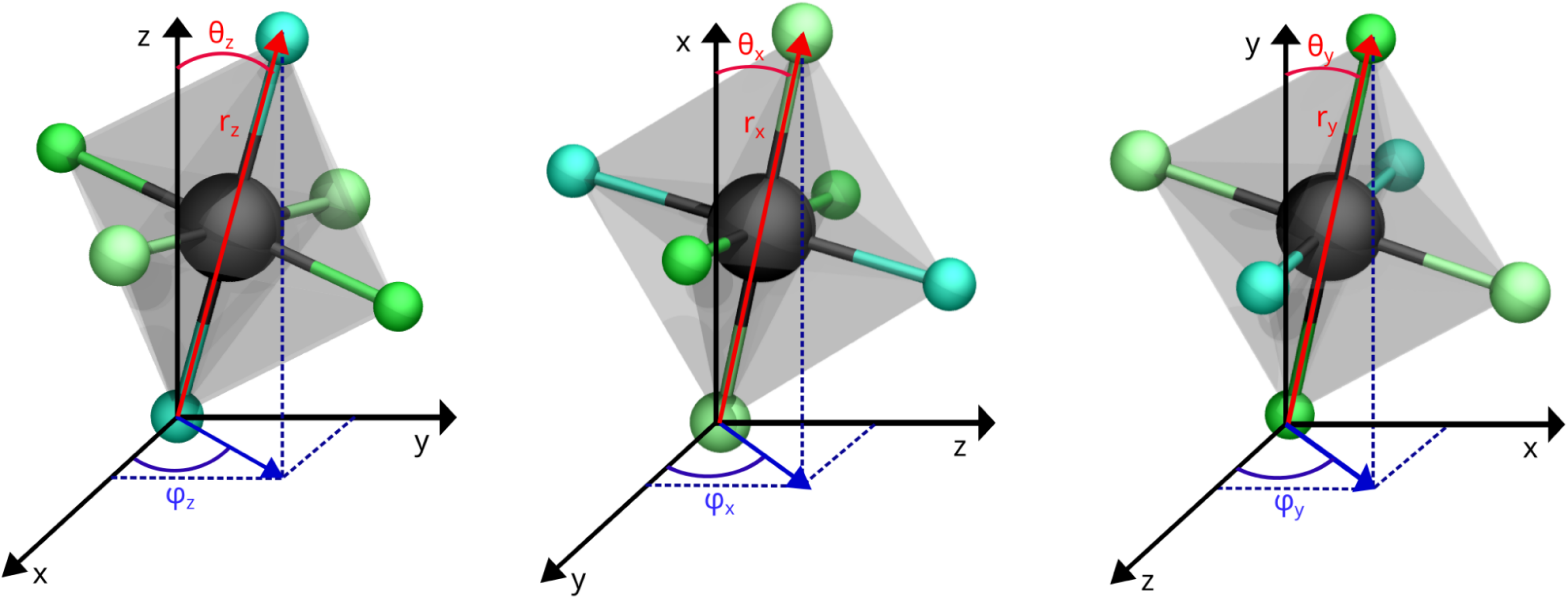
Representation of an octahedron in CsPbBr_3_ using spherical coordinates. From left to right, the panels show the distance vector **r** (red) and the corresponding polar (θ) and azimuthal (φ) angles relative to the *x*, *y*, and *z* axes. The central Pb atom is depicted in gray, while the Br atoms are colored in varying shades of green for visual clarity. All three panels depict the same octahedron, shown from different orientations with respect to each Cartesian axis.

## Results and Discussion

The angular coordinates θ and φ provide a quantitative measure of octahedral rotations and tilts at the atomic scale, offering insight into the mechanisms governing their rearrangements across phases and temperatures. We first examine the ensemble of octahedra to determine the most probable orientations as a function of temperature, capturing the evolution of orientational disorder. We then follow the trajectories of individual octahedra, allowing us to resolve the microscopic processes underlying their rotational and tilting dynamics. This analysis reveals distinct precessional motions that emerge under different thermal conditions.

Figure 4a shows the distribution of the angle θ as a function of temperature. To aid in the analysis, the cooler colors (blue) represent lower temperatures, while the warmer colors (red) indicate higher temperatures. In this analysis, the angle θ was calculated for all octahedra within the supercell and throughout the entire simulation (1 ns). The values obtained align well with prior studies on octahedral tilting in this material, supporting both the accuracy of our methodology and the suitability of the polar coordinate descriptors in capturing the structural behavior.^30,34^ Two distinct features can be identified in the distributions: the first is the broadening of the distribution, which reflects the increase in angular fluctuations with increasing temperature. The second is the reduction of the distribution’s maximum, indicating that with increasing temperature the peak of the distribution shifts toward lower angles, reflecting a tendency of the octahedra to orient closer to the principal axis; however, perfect alignment (zero degrees) has a probability that tends to zero.

**Figure 4:**
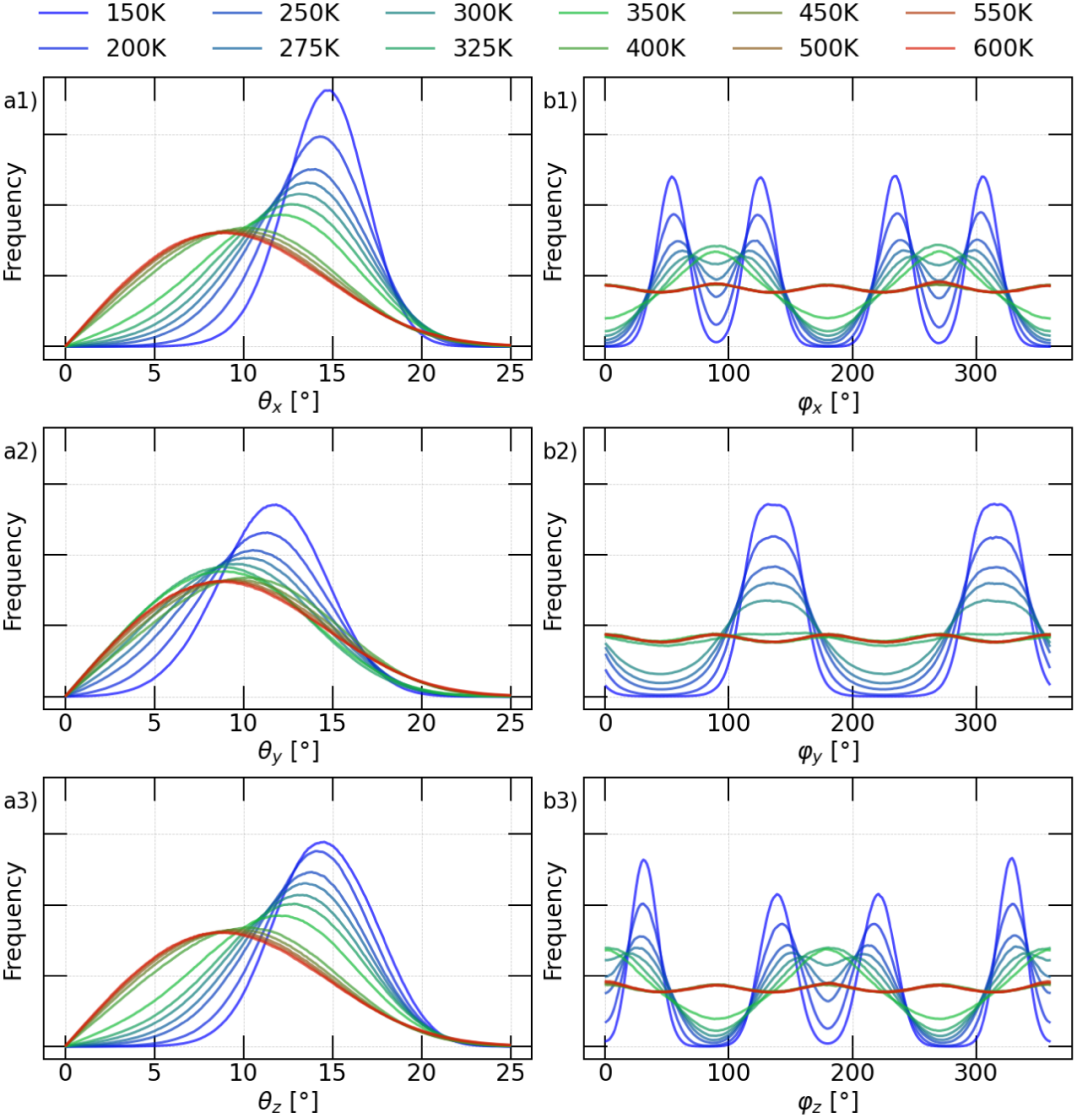
(a) Histogram of the angle θ as a function of temperature, showing the distribution of θ*_x_*, θ*_y_*, and θ*_z_* values corresponding to the three Cartesian directions. (b) Variation of the angle φ distribution as a function of temperature, indicating the values of φ*_x_*, φ*_y_*, and φ*_z_* for the three Cartesian directions.

It is also possible to observe that the distribution maximum is approximately 12° in the *y* direction, compared to 15° in the *x* and *z* directions, reflecting directional differences due to the orthorhombic and tetragonal phases at lower temperatures. As the temperature increases, the distributions along all three directions converge to approximately 9°, and for temperatures above 400 K, the profiles become identical, showing no significant thermal variation. This behavior corresponds to the tetragonal-to-cubic phase transition, and beyond this temperature, the behavior of θ remains similar across all temperatures tested. The convergence of the distributions at high temperatures reflects the effect of the cubic phase symmetry, which renders the angular behavior equivalent along all directions.

Figure 4b shows the distribution of the angles φ_*i*_ (where *i* = *x*, *y*, *z*) as a function of temperature. Similarly to θ, this analysis was performed by evaluating the variations of all octahedra within the supercell throughout the entire simulation. By examining the three Cartesian directions, distinct behaviors can be observed, in contrast to the behavior of θ. For instance, in the analysis of φ_*x*_ and φ_*z*_, four peaks are visible at low temperatures, corresponding to four preferred orientations. As the temperature increases, the four initial peaks gradually merge into two, as the nearest peaks overlap. This change corresponds to the orthorhombic-to-tetragonal phase transition. At higher temperatures (>400 K), the profiles become nearly uniform over the entire 360° range, with only subtle preferences near 0°, 90°, 180°, and 270°. This indicates that all φ angles are visited and sampled with similar probability during the simulation. Lastly, in the *y* direction, a distinct behavior is evident. At low temperatures, two types of octahedra orientation are observed (indicated by two peaks in the distribution). As the temperature increases, the intensity of these peaks gradually diminishes until they vanish at temperatures above 400 K, where the profiles in all three directions become identical. This behavior does not distinguish the orthorhombic-to-tetragonal phase transition, as no clear differences are observed at the transition temperature; however, it does reflect the tetragonal-to-cubic transition at higher temperatures.

This analysis allows us to identify the regions with the highest probability of finding the octahedra as a function of the polar angles θ and φ, as well as to visualize the areas that are less frequently visited during the simulation. To gain a more complete picture of the octahedral dynamics, it is useful to examine whether continuous pathways exist between the most probable regions of the descriptors, as this provides insight into the preferred routes of octahedral tilting. Figure 5 therefore shows the probability distribution in the θ–φ plane for all octahedra over 1 ns of simulation, enabling a direct characterization of their behavior based on these angular descriptors. As an example, Figure 5 focuses on the evolution of the studied octahedron along the *z* direction, while the corresponding results for the *x* and *y* directions are provided in Figures S2 and S3 of the Supporting Information.

**Figure 5:**
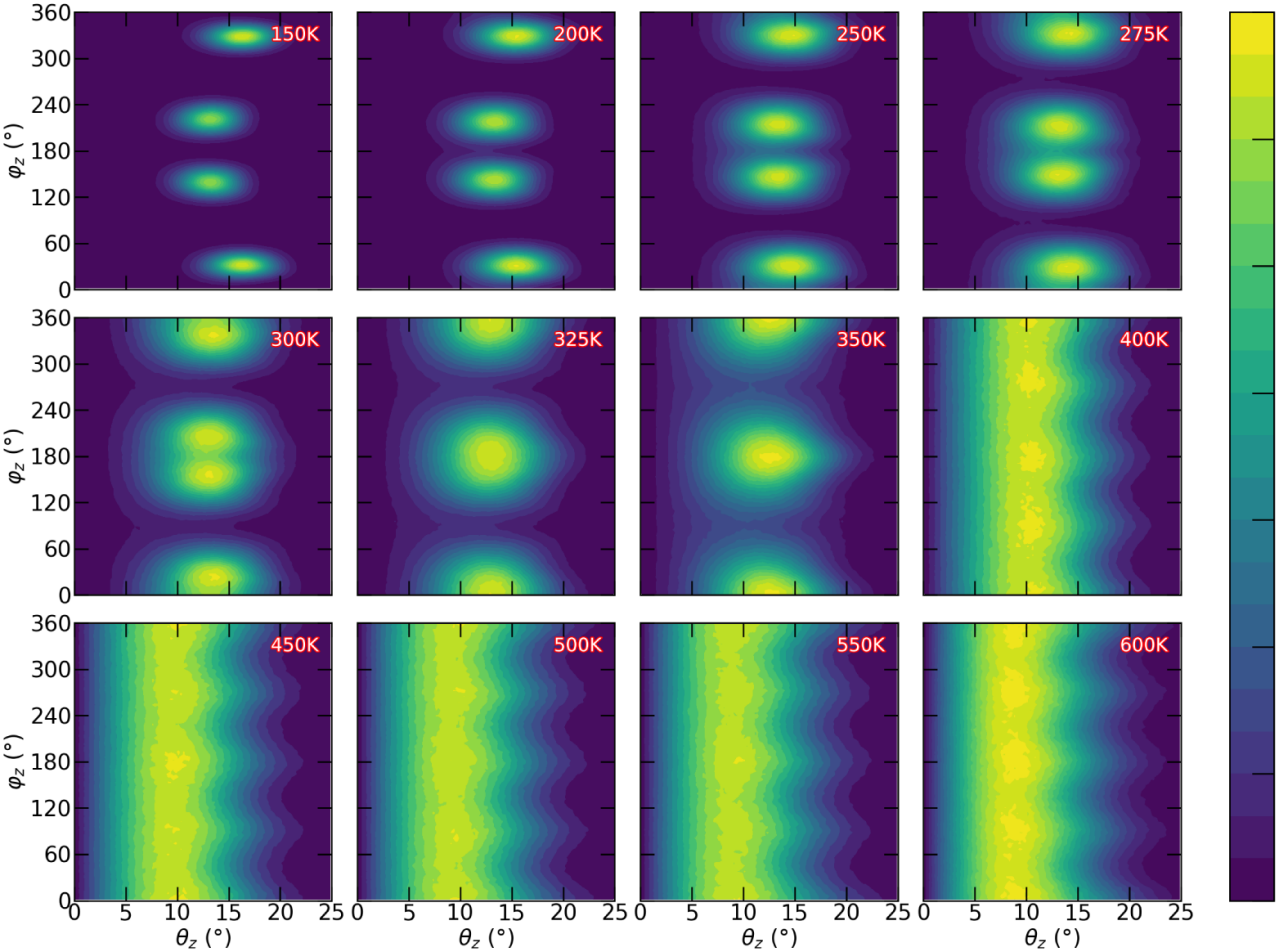
Density map of octahedral trajectories in the θ–φ plane along the *z* direction (θ*_z_*–φ*_z_*) over 1 ns of simulation. The color scale represents the density of visited regions, with higher densities shown in yellow and lower densities in purple. The angles θ*_z_* and φ*_z_* are measured in degrees. This analysis highlights the most frequently visited regions and the areas with less octahedral presence.

Figure 5 presents a more complex pattern when compared to those in Figure 4. This higher level of detail shows that at low temperatures (up to approximately 300 K), four nearly isolated regions of octahedral motion can be distinguished, corresponding to four possible stable states where the octahedra can reside. As the temperature increases, these regions gradually merge into two well-defined zones. At temperatures of 400 K and higher, the regions fully overlap, giving rise to a continuous channel along the φ direction. To further corroborate the information presented in Figure 4a, one can observe that the region of highest occurrence in θ gradually shifts toward lower values as the temperature increases. In addition, the emergence of a continuous channel along the φ direction reflects the nearly uniform probability of occurrence, consistent with the behavior shown in Figure 4b. This is the first evidence of a precession mechanism for the dynamics of the octahedra: they are tilted (θ of around 10 degrees) and they can rotate through all available φ’s.

In the Supporting Information, Figures S2 and S3 present the analyses along the other two Cartesian directions (*y* and *z*). For the *x* case, a similar pattern is observed in the *z* direction, following the same trends. In contrast, the behavior along the *y* direction exhibits notable differences. One key distinction is that the merging of the high-occurrence regions takes place at lower temperatures. Nevertheless, at low temperatures, even up to 325 K, two distinct high-occurrence regions remain identifiable, whereas above 400 K the typical channel-like structure observed in all three directions becomes evident.

The main conclusion from Figures 4 and 5 is that θ, related to the tilting of the octahedra, cannot vary significantly, remaining around a particular value that depends on temperature. On the other hand, φ can be fully explored at high temperatures, indicating rotational dynamics of the octahedra.

To evaluate the generality of this behavior, we performed the same analysis for CsPbI_3_ at 500 and 600 K, temperatures at which the perovskite phase is cubic. The results reveal the same qualitative dynamical features observed for CsPbBr_3_ (Figure 5), namely a restricted distribution in θ and a fully developed rotational channel in φ. The only noticeable difference is that the φ channel in CsPbI_3_ is centered at slightly larger θ values, reflecting a modest shift in the equilibrium tilting amplitude. The corresponding molecular dynamics analysis is presented in Figure S4 of the Supporting Information.

Up to this point, we have provided a general overview of the collective octahedral behavior. However, this type of analysis does not offer direct insights into the temporal evolution of individual octahedra. To address this, we begin by analyzing the trajectory of a single representative octahedron in terms of the θ and φ variables.

### Individual Octahedra Behavior

The most intuitive mechanism for the dynamics of the octahedra, based on the double-well model of the potential energy surface, involves the octahedron moving between the two wells through a configuration aligned with the principal axis, corresponding to the *a* ^+^ ⇒ *a* ^0^ ⇒ *a* ^–^ displacements according to Glazer’s notation. Here we will also evaluate mechanisms associated with precession and nutation. Precession refers to the slow, circular rotation of the octahedron’s axis around a fixed orientation, while nutation corresponds to smaller, oscillatory tilting motions superimposed on the precessional movement. These processes are inherently dynamic, so analyzing the temporal trajectory of each octahedron helps to understand which mechanisms are allowed, which are forbidden, and the frequency of their occurrence. For this purpose, Figure 6 shows, as an example, how an individual octahedron moves throughout the entire simulation. Additionally, the relationship between the θ and φ angles can be clearly observed (a comprehensive analysis of the remaining Cartesian directions is provided in Figures S5 and S6 in the Supporting Information).

**Figure 6:**
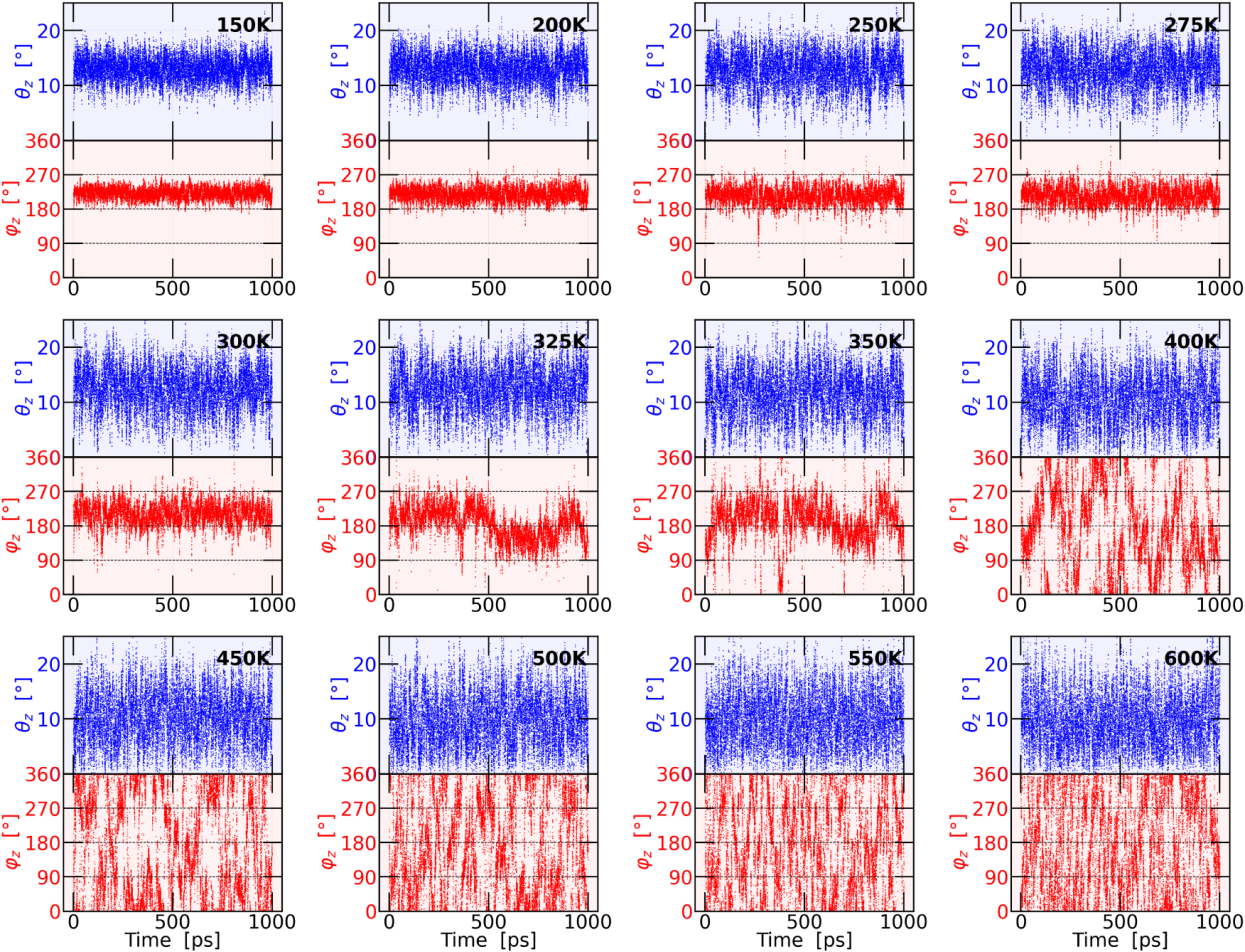
Temporal evolution of the θ*_z_* and φ*_z_* angles for a single octahedron at different temperatures. The variations in φ*_z_* are represented in red, while the variations in θ*_z_* are shown in blue. Each panel is labeled with the corresponding temperature, and black dashed lines indicate the reference angles of φ*_z_* = 90°, 180°, and 270° to enhance visualization.

The analysis of the values of the angle θ (represented in blue in the figure), shows the presence of oscillatory behavior. At low temperatures, the system on average remains away from θ = 0° (taken as a reference since it corresponds to the configuration in which the octahedron is aligned with the Cartesian axis). As the temperature increases, the amplitude of the oscillations grows. However, as shown in Figures 4 and 5, it is unlikely that θ reaches exactly zero. From this analysis, one of the proposed mechanisms becomes evident: the nutation of the octahedron from its “equilibrium” position toward alignment with the principal axis.

Second, analyzing the behavior of φ (red lines) presented in Figure 6, we observe a localized motion at low temperatures, where the octahedron remains confined within one of the regions identified in Figures 4 and 5, corresponding to a specific φ range. As the temperature increases, the octahedron exhibits greater mobility, as reflected by the broader distribution of φ values along its trajectory. At sufficiently high temperatures, some octahedral trajectories complete a full rotation in φ, indicating the presence of precessional motion around the axis. This finding reveals a more complex tilting behavior than the simple *a* ^+^ ⇒ *a* ^0^ ⇒ *a* ^–^ reorientation.

In general, it can be observed that at both low and high temperatures the octahedral motion exhibits oscillatory behavior in both descriptors, with the amplitude increasing as the temperature rises. It is important to emphasize that the octahedral motion does not correspond to a single mechanism of movement; rather, it consists of a combination of motions occurring simultaneously. This arises from the soft nature of the material, where anharmonicities make the dynamics more complex than a simple, idealized behavior.

To quantify and approximate the precession frequency, we analyzed the trajectory of each octahedron throughout the simulation. In this analysis, we identified trajectories in which the angle φ evolves through a full 360° rotation. Figure 7 presents the frequency analysis of these precession events (number of events per unit time, expressed in cm^–1^) for different mobility scales. We evaluated the number of times an octahedron completed either a half rotation of 180° or a full 360° rotation in φ. The study of half rotations (180°) provides insight into how the octahedron transitions from one minimum of the double-well potential to the other, since in φ these energy minima are separated by 180°. In contrast, the analysis of full precession allows us to determine whether the octahedron returns to its initial double-well position or instead completes an entire rotational cycle around the axis. The results shown in Figure 7 correspond to the average values calculated over all octahedra in the perovskite. For both processes, we observe that the trends in the *x* and *z* directions are nearly identical, consistent with the results discussed thus far in this manuscript. In contrast, the behavior in the *y* direction differs from the other directions but converges at approximately 400 K. At this temperature, the overall structure becomes cubic, leading to equivalent behavior in all three directions.

**Figure 7:**
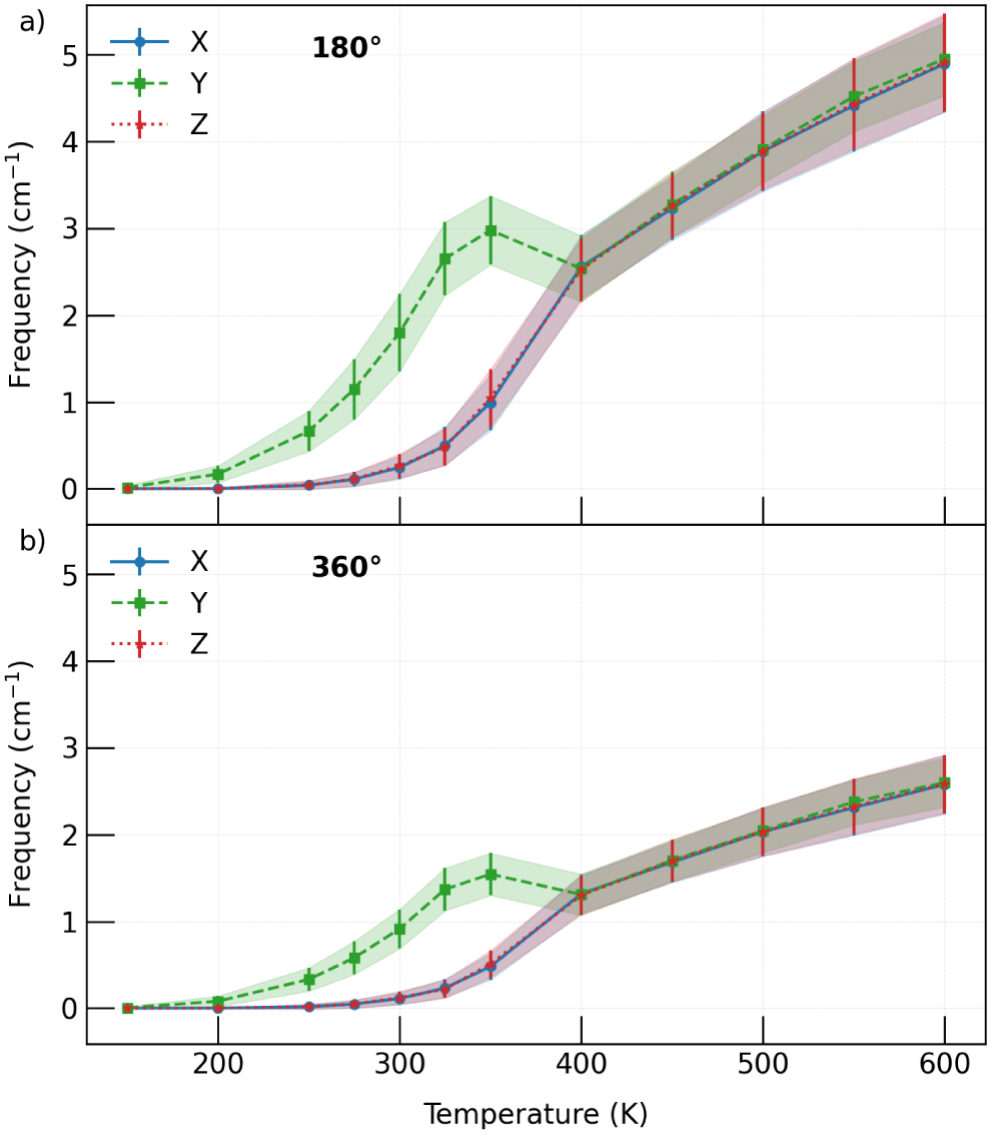
Precession frequencies (cm^–1^) as a function of temperature for the perovskite octahedra. Frequencies are shown along the *x*, *y*, and *z* directions. Each value corresponds to the average over all octahedra in the simulation cell, and the error bars represent the standard deviation of the distribution of independently computed frequencies. The *x* and *z* directions appear superimposed.

Based on the results presented in Figures 6 and 7, we conclude that complete precessional trajectories constitute an important mechanism governing octahedral tilting. This conclusion is supported by the analysis of single-octahedron trajectories (Figure 6), which reveal motion spanning the entire φ range, together with the frequency analysis (Figure 7B), which highlights the crucial role of temperature in this process. In this way, we are able to quantify the occurrence of precession across the different phases exhibited by the perovskite within the studied temperature range. Furthermore, we conclude that the connection between the double-well minima on the potential energy surface is achieved through processes involved in precession, with sufficient freedom to either return to the initial minimum or complete a full precessional cycle.

Here we also aim to evaluate more deeply the possibility of the *a* ^+^ ⇒ *a* ^0^ ⇒ *a* ^–^ mechanism taking place. If such a mechanism were present and analyzed through θ, one would expect to observe regions in the trajectory of individual octahedra (Figure 6) where it decreases, reflecting a reduction in tilting as the octahedron aligns with the principal axis, followed by an increase as it translates to the opposite side of the double well. From the perspective of φ, in an ideal case, the angle should remain constant at a given value until the octahedron aligns with the axis, at which point it would abruptly switch to the corresponding value on the opposite side. This would appear as a discontinuity in the φ descriptor (180°).

When analyzing θ for a representative octahedron (Figure 6), confirming this process with certainty becomes challenging. As discussed previously and illustrated in Figure 6, the motion is predominantly oscillatory, making it difficult to determine whether the octahedron simply aligns and returns to the same minimum (nutation) or instead crosses to the opposite side of the double well through *a* ^+^ ⇒ *a* ^0^ ⇒ *a* ^–^ displacements. The values of θ associated with these two possibilities are too similar to allow an unambiguous distinction.

To resolve this ambiguity, we analyze the step-to-step variation of φ (φ_*t*+1_ – φ_*t*_). We retain only values within ±5° of ±180°, enhancing the identification of abrupt angular changes associated with octahedral reorientation. From this analysis, the mechanism involving the connection of double-well minima through *a* ^+^ ⇒ *a* ^0^ ⇒ *a* ^–^ displacements can thus be ruled out, as the frequency of events occurring within the relevant φ regions is orders of magnitude lower than that associated with precessional motion. This conclusion is consistent with previous results showing that the zero-tilting configuration (*a* ^0^ *a* ^0^ *a* ^0^) corresponds to a maximum of the potential energy surface and is therefore rarely sampled.^23^

To further elucidate the origin of the observed dynamics, we analyze the joint distribution, *P*_0_, of the collective variables (θ, φ), which characterize the magnitude and orientation of the octahedral tilts (Figure 5). and construct an effective free-energy-like surface defined as *G* ∼ −*k*_*B*_ *T*ln*P*_0_(θ, φ), where *k*_*B*_ is the Boltzmann constant and *T* is the equilibrium simulation temperature. Although this quantity does not represent an equilibrium free energy, it provides a meaningful description of the regions of configuration space dynamically explored by the system.^54,55^ To visualize this landscape, we focus on the temperature *T* = 400 K, at which precessional motion is already clearly established. The effective surface, shown in Figure 8, is presented as a projection onto the (θ, φ) plane (top panel) and as a three-dimensional representation (bottom panel). It exhibits a Mexican-hat-like character with a very flat energy landscape along φ, explaining the origin of the precessional motion and confirming the soft, anharmonic nature of the material.

**Figure 8:**
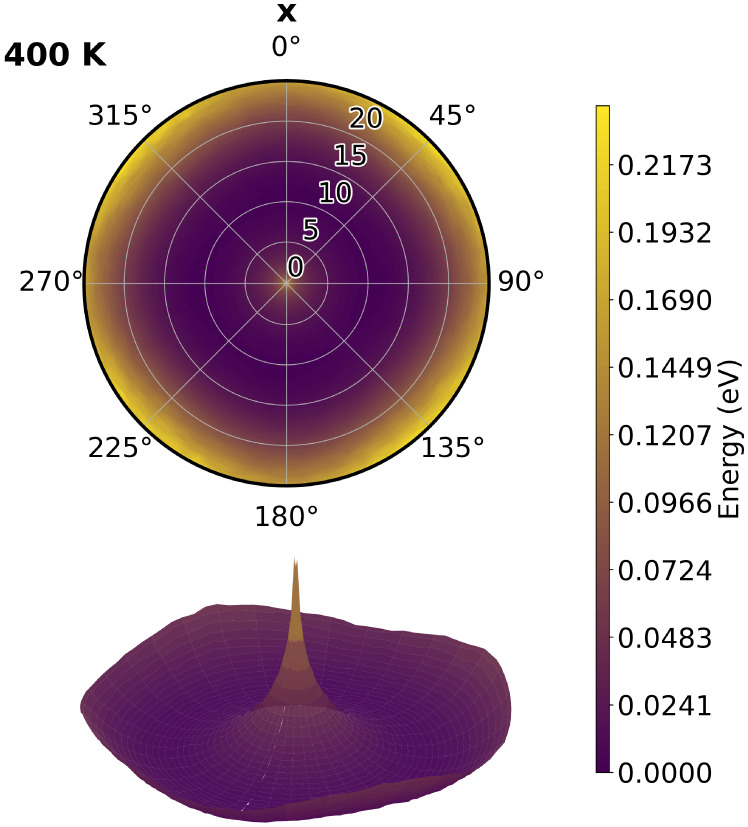
Effective free-energy-like surface *G* ∼ −*kT*ln*P*_0_(θ, φ) at *T* = 400 K along the *x* direction. Top: projection onto the (θ, φ) plane; bottom: corresponding three-dimensional representation. The radial coordinate encodes θ, while the polar angle encodes φ.

## Conclusions

In this work, we have investigated the dominant dynamic mechanisms of octahedra in CsPbBr_3_ using angular descriptors. We have built a precise machine learning potential for this compound, and our analysis indicates that the intuitive *a* ^+^ ⇒ *a* ^0^ ⇒ *a* ^–^ displacement mechanism does not constitute a statistically significant contribution to octahedral dynamics under the conditions explored in this work, consistent with the energy landscape described in previous studies.^23^

The predominant dynamic behavior arises from a combination of precessional and nutational motions, resulting in complex, multicomponent, and temperature-dependent octahedral dynamics. At low temperatures, φ fluctuations are limited and θ remains small, whereas at higher temperatures, both angles exhibit broader excursions due to enhanced thermal mobility. These findings emphasize the importance of analyzing both angular descriptors to capture the full spectrum of octahedral dynamics across different phases.

Overall, this study provides strong evidence that octahedral motion in CsPbBr_3_ is governed primarily by precessional dynamics. Additional simulations for CsPbI_3_ show qualitatively similar coupled precession–nutation behavior, suggesting that this mechanism may also be relevant in related halide perovskites with comparable structural motifs. This framework offers a comprehensive understanding of temperature-dependent structural fluctuations in halide perovskites and highlights the utility of angular descriptors in capturing complex atomic-scale behaviors.

## Supplementary Material



## Notes

Details of deep neural network potential construction and validation; parity plots comparing DFT and neural network energies and forces; angular density maps of octahedral trajectories in the θ–φ plane; transferability analysis to CsPbI_3_; temperature-dependent temporal evolution of octahedral angular variables (PDF)
