# Supplementary material for: Precessional Dynamics of Octahedra in CsPbBr3
Source: J Chem Theory Comput. 2026 Apr 16;22(8):4125–35. doi: 10.1021/acs.jctc.5c02166 (PMC13130863; doi:10.1021/acs.jctc.5c02166)
Supplement: Supplementary file 1 [file ct5c02166_si_001.pdf]

Supporting Information for

Precessional Dynamics of Octahedra in $CsPbBr_3$

Lucas Martin Farigliano*[1,2], Márcio S. Gomes-Filho[3], Alexandre Reily Rocha[4], and Gustavo Martini Dalpian*[1]

[1]Departamento de Física dos Materiais e Mecânica, Instituto de Física, Universidade de São Paulo, São Paulo 05508-090, São Paulo, Brazil
[2]INFIQC, CONICET, Departamento de Química Teórica y Computacional, Facultad de Ciencias Químicas, Universidad Nacional de Córdoba, Argentina
[3]Centro de Ciências Naturais e Humanas, Universidade Federal do ABC, 09210-580 Santo André, SP, Brazil
[4]Institute of Theoretical Physics, São Paulo State University, Campus São Paulo,01140-070, Brazil

*Email: lucmfari@if.usp.br,dalpian@if.usp.br

Supporting Information

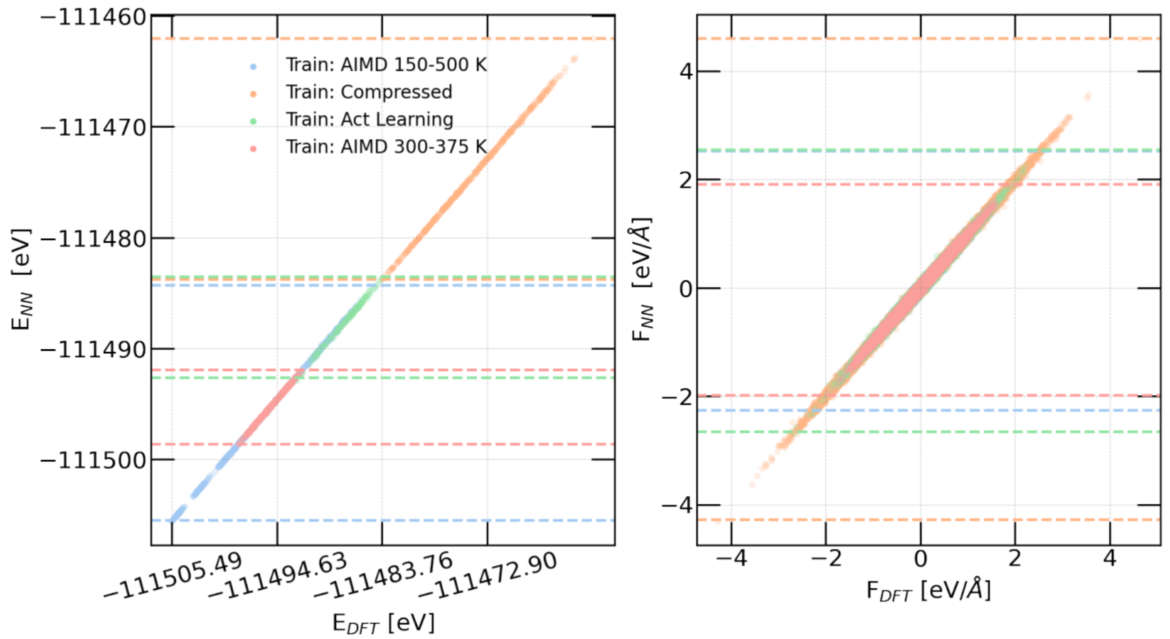

Figure S1: Deep neural network potential quality assessment: A) Potential energy and B) the forces predicted by the neural network (E_{NN} and F_{NN}) compared to the DFT reference values (E_{DFT} and F_{DFT}).

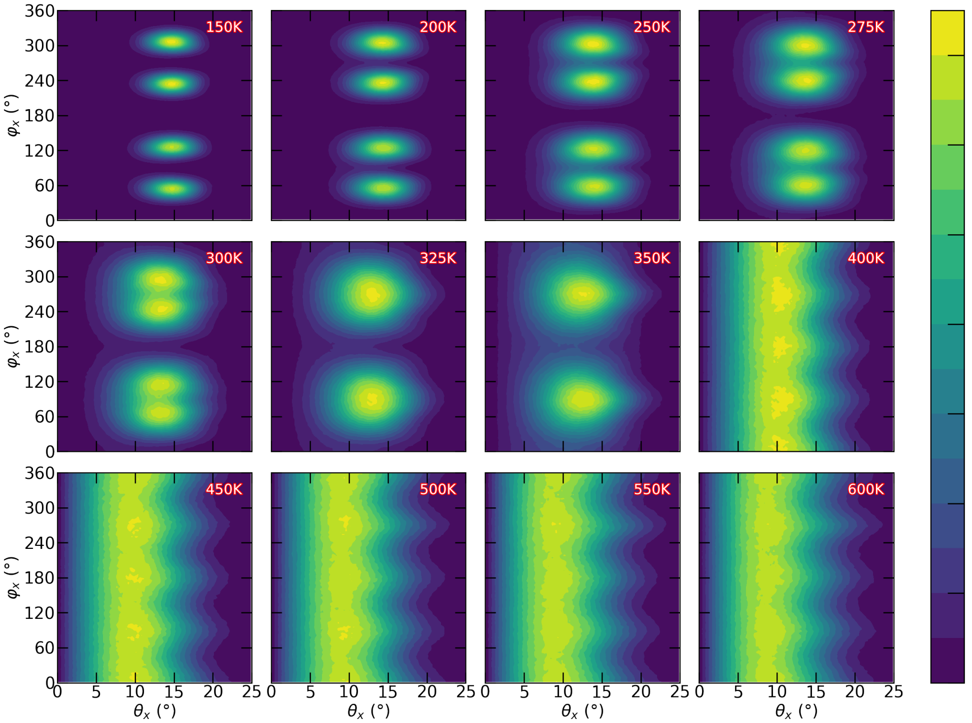

Figure S2: Density map of octahedral trajectories in the θ-φ plane along the x direction (θ_x-φ_x) over 1 nanosecond of simulation. The color scale represents the density of visited regions, with higher densities shown in yellow and lower densities in purple. The angles θ_x and φ_x are measured in degrees. This analysis highlights the most frequently visited regions and the areas with less octahedral presence.

To evaluate the transferability of the angular dynamical framework discussed for $CsPbBr_3$, we constructed a deep neural network interatomic potential for $CsPbI_3$ employing exactly the same DeepMD-kit implementation, DP-SE descriptor, network architecture, and hyperparameters used for $CsPbBr_3$, ensuring full methodological consistency.

The reference DFT dataset was generated using the same computational infrastructure and CP2K setup described for $CsPbBr_3$. An initial pool of 1482 configurations was extracted from *ab initio* molecular dynamics simulations performed at different pressures. To improve the sampling of thermally activated distortions and anharmonic fluctuations, four iterations of active learning were carried out, involving temperature ramps between 100 K and 650 K under multiple pressure conditions. During this concurrent learning procedure, 270 additional configurations were selected based on force-model deviation criteria and subsequently labeled at the DFT level. The final training dataset therefore comprised a total of 1752 configurations.

The resulting DP model shows high accuracy with respect to the DFT reference data. On the training set, the root mean squared error (RMSE) is 0.00041 eV/atom for energies and 0.029 eV/Å for atomic forces. These values are comparable to those obtained for $CsPbBr_3$ and confirm the reliability of the potential for describing the structural and dynamical distortions of $CsPbI_3$.

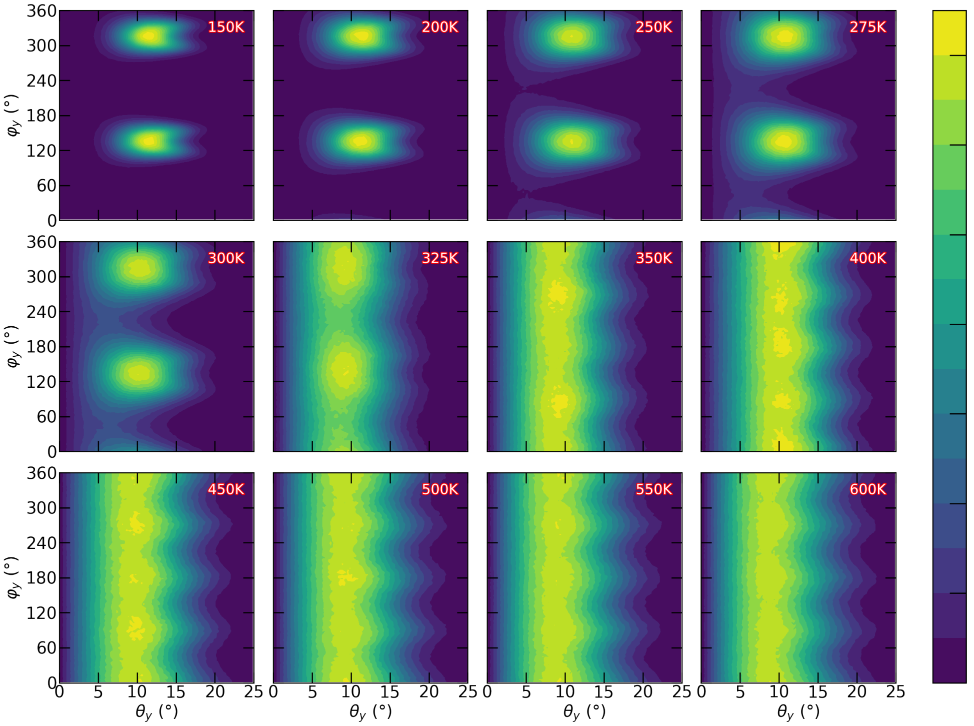

Figure S3: Density map of octahedral trajectories in the θ-φ plane along the y direction (θ_y-φ_y) over 1 nanosecond of simulation. The color scale represents the density of visited regions, with higher densities shown in yellow and lower densities in purple. The angles θ_y and φ_y are measured in degrees. This analysis highlights the most frequently visited regions and the areas with less octahedral presence.

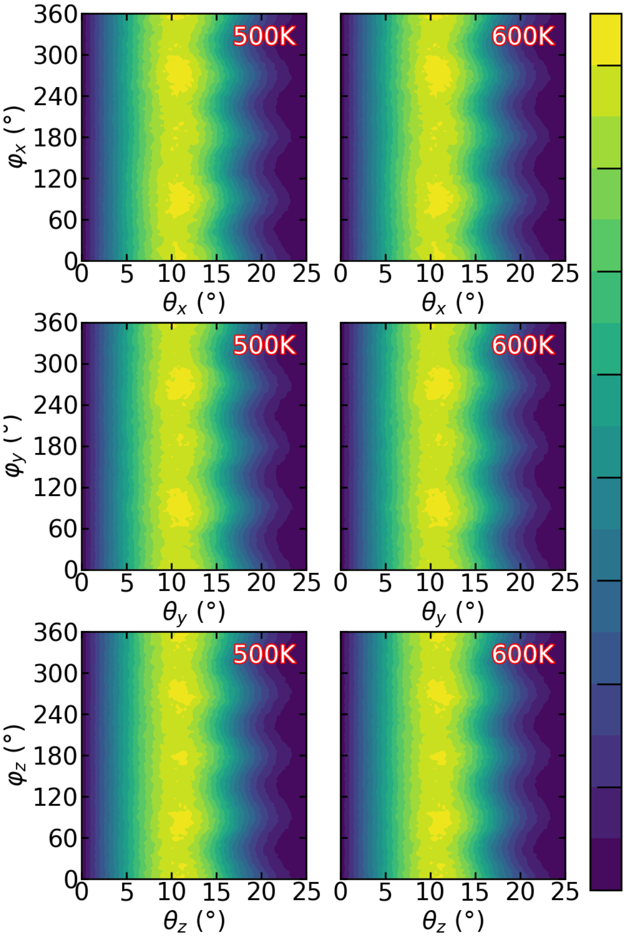

Figure S4: Density map of octahedral trajectories in the θ-φ plane over 1 nanosecond of simulation for $CsPbI_3$. The color scale represents the density of visited regions, with higher densities shown in yellow and lower densities in purple. The angles are measured in degrees. This analysis highlights the most frequently visited regions and the areas with less octahedral presence.

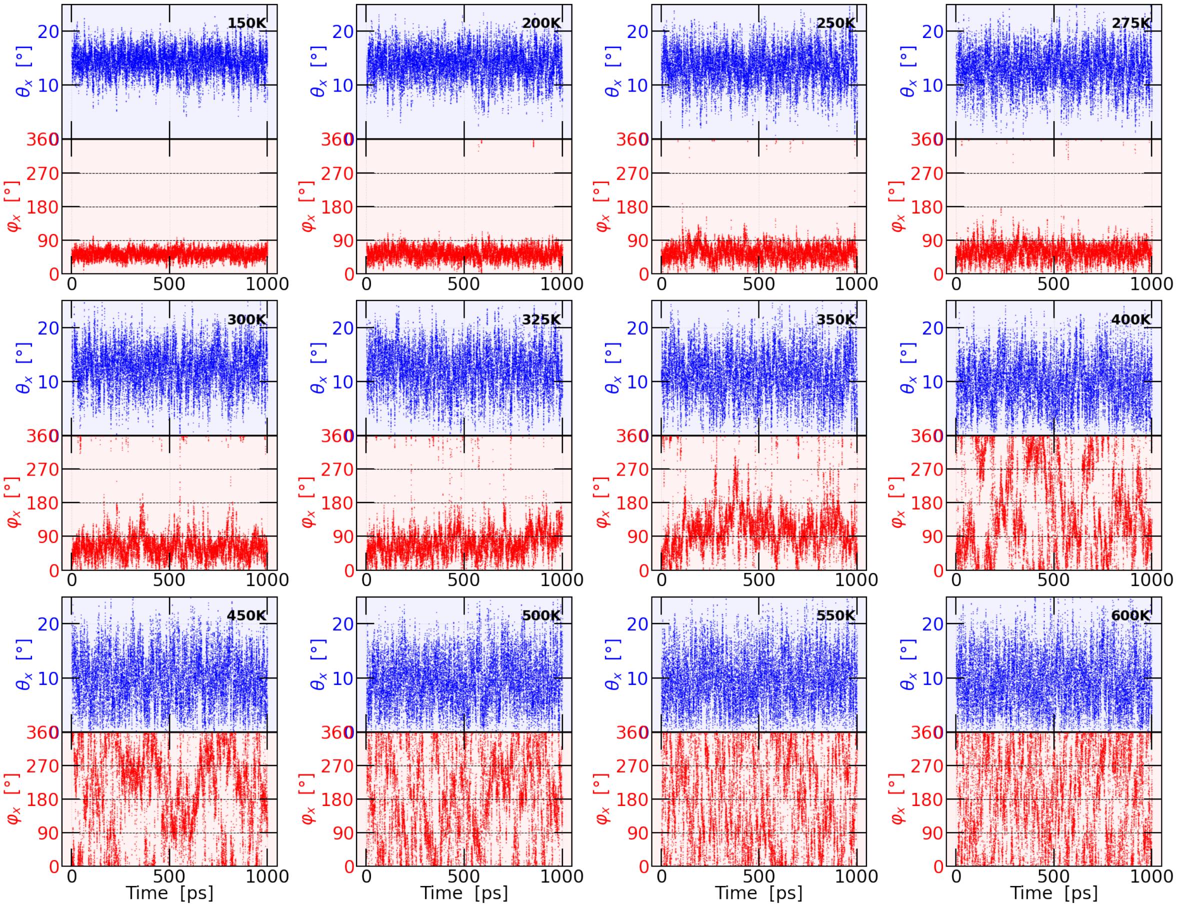

Figure S5: Temporal evolution of the θ_x and φ_x angles for a single octahedron at different temperatures. The variations in φ_z are represented in red, while the variations in θ_x are shown in blue. Each panel is labeled with the corresponding temperature, and black dashed lines indicate the reference angles of $\varphi_x = 90°$, 180°, and 270° to enhance visualization.

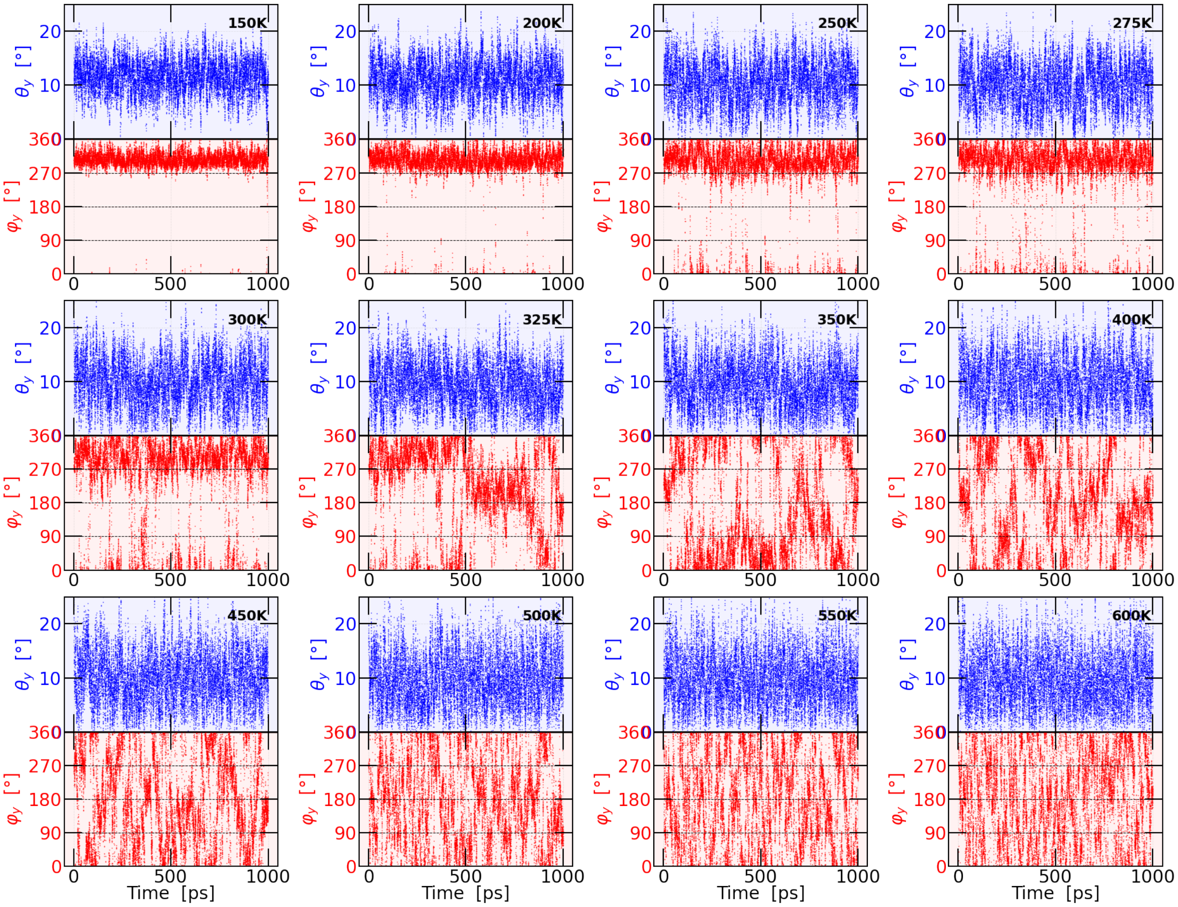

Figure S6: Temporal evolution of the θ_y and φ_y angles for a single octahedron at different temperatures. The variations in φ_y are represented in red, while the variations in θ_y are shown in blue. Each panel is labeled with the corresponding temperature, and black dashed lines indicate the reference angles of $\varphi_y = 90°$, 180°, and 270° to enhance visualization.
